# Biomarker characterization of clinical subtypes of Parkinson Disease

**DOI:** 10.1038/s41531-022-00375-y

**Published:** 2022-08-29

**Authors:** Xiao Deng, Seyed Ehsan Saffari, Nan Liu, Bin Xiao, John Carson Allen, Samuel Yong Ern Ng, Nicole Chia, Yi Jayne Tan, Xinyi Choi, Dede Liana Heng, Yew-long Lo, Zheyu Xu, Kay-Yaw Tay, Wing-Lok Au, Adeline Ng, Eng-King Tan, Louis C. S. Tan

**Affiliations:** 1 Department of Neurology, National Neuroscience Institute, Singapore, Singapore; 2 Duke-NUS Medical School, Singapore, 8 College Rd, Singapore, 169857 Singapore; 3 Centre for Quantitative Medicine, Duke-NUS Medical School, Singapore, Singapore; 4 Programme in Health Services and Systems Research, Duke-NUS Medical School, Singapore, Singapore

**Keywords:** Parkinson's disease, Prognostic markers

## Abstract

The biological underpinnings of the PD clusters remain unknown as the existing PD clusters lacks biomarker characterization. We try to identify clinical subtypes of Parkinson Disease (PD) in an Asian cohort and characterize them by comparing clinical assessments, genetic status and blood biochemical markers. A total of 206 PD patients were included from a multi-centre Asian cohort. Hierarchical clustering was performed to generate PD subtypes. Clinical and biological characterization of the subtypes were performed by comparing clinical assessments, allelic distributions of Asian related PD gene (SNCA, LRRK2, Park16, ITPKB, SV2C) and blood biochemical markers. Hierarchical clustering method identified three clusters: cluster A (severe subtype in motor, non-motor and cognitive domains), cluster B (intermediate subtype with cognitive impairment and mild non-motor symptoms) and cluster C (mild subtype and young age of onset). The three clusters had significantly different allele frequencies in two SNPs (Park16 rs6679073 A allele carriers in cluster A B C: 67%, 74%, 89%, *p* = 0.015; SV2C rs246814 T allele distribution: 7%, 12%, 25%, *p* = 0.026). Serum homocysteine (Hcy) and C-reactive protein (CRP) levels were also significantly different among three clusters (Mean levels of Hcy and CRP among cluster A B C were: 19.4 ± 4.2, 18.4 ± 5.7, 15.6 ± 5.6, adjusted *p* = 0.005; 2.5 ± 5.0, 1.5 ± 2.4, 0.9 ± 2.1, adjusted *p* < 0.0001, respectively). Of the 3 subtypes identified amongst early PD patients, the severe subtype was associated with significantly lower frequency of Park16 and SV2C alleles and higher levels of Hcy and CRP. These biomarkers may be useful to stratify PD subtypes and identify more severe subtypes.

## Introduction

Parkinson’s disease (PD) is the most common hypokinetic movement disorder with significant heterogeneity in symptoms and outcomes. Non-Motor symptoms (NMS) resulting from various neurotransmitter pathway dysfunctions affects both the central and peripheral nervous systems, which contribute to PD heterogeneity^1,2^. Subtype identification has been established as one of the top three clinical research priorities in the field of PD^3^. Identification of PD subtype could be valuable in revealing the underlying etiology and understanding the disease course. More importantly, PD subtyping could guide the design of clinical trial and future personalized PD treatment.

Cluster analysis, a data-driven approach could help to define the disease phenotypes. Most studies use cluster analysis to stratify PD subtypes based on clinical data, such as motor, NMS and demographic features^4,5^. These studies have however been limited by the inclusion of PD patients from different disease stages; absence of genetic data that may influence clinical heterogeneity; and limited analysis of Asian cohorts.

The biological underpinnings of the PD clusters remain unknown as the existing multidimensional data-driven derivation of PD clusters lacks biomarker characterisation. PD biomarkers including clinical, blood, cerebrospinal fluid (CSF) and imaging biomarkers have been playing increasingly important roles in early diagnosis and disease prognostication^6^. Blood biomarkers may have wider implications than CSF and imaging biomarkers as they are more accessible at lower cost^7^.

Homocysteine (Hcy) and C-reactive protein (CRP) are two blood biochemical biomarkers that are associated with PD severity. Severe PD subtypes have been found to have significantly higher levels of CRP^8^ while elevated plasma Hcy level was found in depressed and cognitively impaired PD patients^9^. Vitamin D, uric acid(UA) and lipids are thought to play important neuroprotective roles in PD. Studies have shown that higher serum UA levels were associated with a lower risk of PD development^10^ and more benign prognosis in PD patients^11^. Vitamin D deficiency is common in PD patients^12^ and lower vitamin D levels have been associated with worse prognosis in PD^13^. In addition, serum lipid biomarkers were reduced in PD patients compared to healthy controls^14–17^.

To understand the clinical heterogeneity of PD, we used cluster analysis to search for subtypes in a multi-centre, Asian early PD cohort. The aims of our study are: (1) To identify distinct PD clusters from a comprehensive dataset; (2) To provide clinical and biological features of the identified subtypes by comparing the clinical characteristics, allelic distributions of Asian related PD genes, and blood biochemical markers.

## Results

### Patient demographic and clinical characteristics

A total of 206 PD patients were enrolled in the study and 122 (59.2%) patients were male. Mean age of diagnosis was 63.5 ± 9.0 years. MCI was presented in 108 (52.4%) patients at baseline in our cohort. A summary of patient demographics and clinical characteristics was shown in Table 1. The comparison of comorbidities among three clusters can be found in Supplementary Table 1.

**Table 1:** Patient demographics and clinical characteristics.

Variable	Whole cohort (*n* = 206)
Sex: Male	122 (59.2%)
Ethnicity:	
Chinese	177 (85.9%)
Malay	11 (5.3%)
Indian	14 (6.8%)
Others	4 (2%)
Education (Year)	10.6 ± 4.4
Age of diagnosis (year)	63.5 ± 9.0
PRS	0.7 (0.3–1.0)
Patients with RBD symptoms	51 (24.8%)
MCI	108 (52.4%)
Memory score	−1.0 (−1.9–0.2)
Visuospatial score	−0.5 (−1.2–0.2)
Attention score	−0.3 (−0.9–0.3)
Language score	−0.1 (−1.0–0.3)
Executive score	−0.2 (−0.8–0.4)
MDS-UPDRS part I score	3 (2–6)
MDS-UPDRS part II score	3 (1–6)
MDS-UPDRS part III score	20 (15–26)
PIGD score	1 (1–2)
Tremor score	3 (1–5)
HADS Anxiety Total score	2 (0–4)
HADS Depression Total score	2 (1–4)
NMSS Total score	14 (9–26)
LEDD	194.8 ± 138.0

Categorical variables reported as frequency (%); continuous variables reported as mean ± standard deviation or median and first and third quartile, where appropriate.

*PRS* polygenic risk score, *RBD* rapid eye movement sleep behaviour disorder, *MCI* mild cognitive impairment, *MDS-UPDRS* movement disorder society-unified Parkinson’s disease rating scale, *PIGD* postural instability and gait disorder, *HADS* Hospital Anxiety Depression Scale, *NMSS* Non-motor symptom scale, *LEDD* levodopa equivalent daily dose.

### Cluster analysis results

**Fig. 1:**
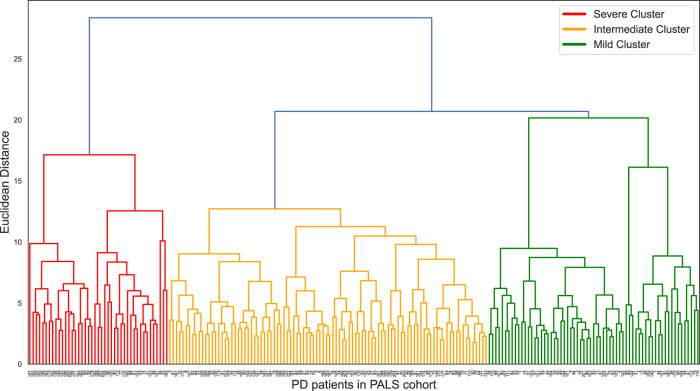
Dendrogram of the final hierarchical cluster solution in the PALS cohort. Reference. https://www.analyticsvidhya.com/blog/2019/05/beginners-guide-hierarchical-clustering/.

Three independent PD clusters were identified from hierarchical cluster analysis (Fig. 1). The features comparison among three clusters can be seen in Table 2. The three clusters had significant differences in age of diagnosis (mean age of diagnosis of cluster A, B, C was 69.6 ± 7.9, 63.6 ± 7.4, 59.4 ± 9.7 years, *p* < 0.001, respectively). The three clusters also differed significantly in all cognitive domain scores, most of the motor scores (MDS-UPDRS part II, III score, tremor score, PIGD score) and most NMS items (MDS-UPDRS part I score, systolic BP drop, ESS total score and HADS depression score). There were no significant differences in terms of PRS, HADS anxiety score and RBD1Q among the three clusters.

**Table 2:** Cluster features comparison.

Variables included for clustering	Cluster A	Cluster B	Cluster C	*p* value^*^
	Severe cluster	Intermediate	Mild cluster	
	*n* = 43	*n* = 98	*n* = 65	
Age of diagnosis (year)	69.6 ± 7.9	63.6 ± 7.4	59.4 ± 9.7	<0.001
PRS	0.7 (0.3–0.8)	0.7 (0.5–0.8)	0.7 (0.5–1.0)	0.100
Standardized Memory score	−1.8 (−2.6 to −0.9)	−1.3 (−2.0 to −0.7)	−0.1 (−0.6 to 0.3)	<0.001
Standardized Visuospatial score	−1.1 (−1.9 to 0.1)	−0.6 (−1.3 to −0.0)	−0.0 (−0.5 to 0.5)	<0.001
Standardized Attention score	−0.7 (−1.1 to −0.1)	−0.6 (−0.9 to 0.0)	0.3 (−0.4 to 0.9)	<0.001
Standardized Language score	−1.0 (−1.6 to −0.1)	−0.4 (−1.2 to 0.1)	0.3 (−0.0 to 0.8)	<0.001
Standardized Executive score	−0.7 (−1.5 to −0.2)	−0.4 (−0.9 to 0.2)	0.5 (0.1–1.0)	<0.001
MDS-UPDRS part I score	6 (3–8)	2.5 (1–4)	4 (2–7)	<0.001
MDS-UPDRS part II score	6 (4–9)	2 (1–4)	3 (1–6)	<0.001
MDS-UPDRS part III score	32 (26–37)	19.5 (15–24)	18 (14–20)	<0.001
PIGD score	3 (2–4)	1 (1–2)	1 (1–2)	<0.001
Tremor score	5 (3–8)	2 (0–4)	2 (0–3)	<0.001
Orthostatic SBP drop >10 mmhg (%)	15 (35%)	21 (21%)	7 (11%)	0.010
ESS Total Score	9 (5–12)	5 (2–8)	5 (3–7)	<0.001
HADS Anxiety score	2 (1–4)	1 (0–3)	2 (0–6)	0.055
HADS Depression score	4 (2–7)	2 (1–3)	2 (1–4)	<0.001
RBD1Q	0 (0–1)	0 (0-0)	0 (0–1)	0.520

All the variables were standardized before cluster analysis.

Categorical variables reported as frequency (%); continuous variables reported as mean ± standard deviation or median and first and third quartile (where appropriate)

^*^Chi-square or Fisher exact test (where appropriate) for categorical variables, One-way ANOVA or Kruskal Wallis test for continuous variables (depends on normality assumption).

*PRS* polygenic risk score, *MDS-UPDRS* Movement Disorder Society-Unified Parkinson’s Disease Rating Scale, *PIGD* postural instability and gait disorder, *SBP* systolic blood pressure, *ESS* Epworth sleepiness scale, *HADS* Hospital Anxiety Depression Scale, *NMSS* non-motor symptom scale, *RBD1Q* rapid eye movement sleep behaviour disorder single-question screen.

Cluster A was severe subtype in motor, NMS and cognition, which comprised 43 (20.9%) PD patients. Cluster A was the most severe in terms of motor, NMS and cognition domains as supported by the highest MDS-UPDRS part I, II, III scores, PIGD score, cognitive domain scores and depression score. Significant BP drop was the most common in cluster A (35% of the patients in cluster A had significant BP drop vs 21% and 11% in clusters B and C, *p* = 0.010).

The second cluster (cluster B) was the largest cluster with 98 subjects, consisting of 47.6% of PD patients. Cluster B was the intermediate subtype characterized by cognitive impairment with mild NMS. Cognitive domain scores in this cluster were moderate, ranging between cluster A and C. However, patients in this cluster had very mild NMS impairment supported by the lowest MDS-UPDRS part I score (2.5 in cluster B vs 6 and 4 in cluster A and C, *p* < 0.001).

There were 65 patients in cluster C, accounting for 31.6% of the PD patients. Cluster C was a mild subtype with a younger age of onset. The mean age of diagnosis of cluster C was significantly younger than the other two clusters (59.4 ± 9.7 in cluster C vs 63.6 ± 7.4, 69.6 ± 7.9 in cluster B and A, *p* < 0.001). Cluster C had good cognitive performance supported by the highest cognitive domain scores. Significant BP drop was not common in the cluster, with only 7 patients having significant systolic blood pressure drop. The other non-motor and motor profiles in cluster C were relatively mild.

### Characterization of PD subtypes using clinical biomarkers

Clinical variables that were not included in the clustering model were used for post-hoc comparison among the clusters (Table 3). The 3 clusters remained significantly different with regard to MCI rate, MoCA Score, and most of the NMSS domain scores, except domain 4 (perceptual problems) and domain 8 (sexual function), after correction for multiple comparison.

**Table 3:** Post hoc comparison of the baseline demography and clinical features among the three PD clusters.

Post-hoc comparison of other variables	Cluster A	Cluster B	Cluster C	*p* value^*^	*q* value**
	severe cluster	intermediate	mild cluster		
	*n* = 43	*n* = 98	*n* = 65		
Sex: male	29 (67%)	56 (57%)	37 (57%)	0.46	0.4600
MCI	35 (81%)	63 (64%)	10 (15%)	<0.001	<0.0019
MoCA Score	23 (19–26)	25 (22–27)	28 (26–29)	<0.001	<0.0019
NMSS Total Score	26 (14–43)	11 (6–18)	16 (9–32)	<0.001	<0.0019
NMSSD1Score (cardiovascular)	0 (0–4)	0 (0–0)	0 (0–0)	0.003	0.0050
NMSSD2Score (sleep/fatigue)	4 (0–8)	0 (0–3)	1 (0–5)	<0.001	<0.0019
NMSSD3Score (mood/apathy)	1 (0–5)	0 (0–0)	0 (0–2)	<0.001	<0.0019
NMSSD4Score (perceptual problems)	0 (0–0)	0 (0–0)	0 (0–0)	0.27	0.2893
NMSSD5Score (attention/memory)	1 (0–4)	0 (0–1)	0 (0–2)	0.027	0.0338
NMSSD6Score (gastrointestinal)	2 (0–7)	0 (0 - 2)	0 (0–3)	0.004	0.006
NMSSD7Score (urinary)	5 (4–12)	4 (0–5)	2 (0–5)	<0.001	<0.0019
NMSSD8Score (sexual function)	0 (0–2)	0 (0–0)	0 (0–0)	0.26	0.2893
NMSSD9Score (miscellaneous)	0 (0–3)	0 (0–3)	1 (0–4)	0.024	0.0327
LEDD	205.5 ± 103.5	211.3 ± 142.3	162.5 ± 147.8	0.089	0.1458

Categorical variables reported as frequency (%); continuous variables reported as mean ± standard deviation or median and first and third quartile (where appropriate)

^*^Chi-square or Fisher exact test (where appropriate) for categorical variables, One-way ANOVA or Kruskal Wallis test for continuous variables (depends on normality assumption)

**False discovery rate (FDR) method was performed and *q* values were calculated to control for multiple testing and the threshold of *q* values was set as 0.1.

*MCI* mild cognitive impairment, *H&Y* Modified Hoehn and Yahr (H&Y) staging scale, *NMSS* non-motor symptom scale, *LEDD* levodopa equivalent daily dose.

Cluster A consistently had significantly worse performance in all profiles, including highest MCI percentage (81%) and NMSS total score. Cluster B had obvious cognitive impairment with mild NMS and was characterized by having a moderate percentage of MCI (64%) and the lowest NMSS total score (11 vs 26 and 16 in cluster A and C, *p* < 0.001, *q* < 0.0019). Cluster C was a mild subtype and was characterized by having the lowest MCI percentage (15%, 64%, 81%, for cluster C, B, A respectively, *p* < 0.001, *q* < 0.0019).

### Characterization of PD subtypes using blood biomarkers

#### Allelic distributions of Asian related PD genes in three PD clusters

A total of 206 PD patients were genotyped. The *Park16 rs6679073* A allele frequency was 76.7% (158 A allele carriers, including 77 patients carried AA and 81 patients harboured AC), the *SV2C rs246814* T allele frequency was 15.0 % (31 T allele carriers, including 2 patient carried TT and 29 patients carried TC).

The three clusters had significantly different effect allele frequency in these two SNPs (distribution of Park16 rs6679073 A allele carriers in cluster A B C: 67%, 74%, 89%, *p* = 0.015, *q* = 0.065; SV2C rs246814 T allele distribution: 7%, 12%, 25%, *p* = 0.026, *q* = 0.065; Table 4). Cluster A (severe subtype in motor, NMS and cognitive domains) had the lowest percentage of both Park16 and SV2C effect allele, while cluster C (mild subtype and young age of onset) contained the largest number of the carriers of these two SNPs.

**Table 4:** Allelic distributions of Asian related PD genes among the three PD clusters.

SNP	Effect allele	Cluster A	Cluster B	Cluster C	*p* value*	*q* value**
		severe cluster	intermediate	mild cluster		
		*n* = 43	*n* = 98	*n* = 65		
*Park16 rs6679073*	A	29 (67%)	72 (74%)	57 (89%)	0.015	0.065
*SV2C rs246814*	T	3 (7%)	12 (12%)	16 (25%)	0.026	0.065
*SNCA rs6826785*	C	36 (86%)	77 (86%)	51 (82%)	0.87	0.880
*Lrrk2 rs141336855*	T	1 (2%)	3 (3%)	3 (5%)	0.88	0.880
*ITPKB rs16846351*	G	6 (15%)	10 (11%)	5 (9%)	0.60	0.880

^*^Fisher’s exact test was carried out to compare the Gene allelic distributions among different clusters.

**False discovery rate (FDR) method was performed and *q* values were calculated to control for multiple testing and the threshold of *q* values was set as 0.1.

#### Comparison of blood biochemical markers among three clusters

We found significant differences in Hcy and CRP levels among three clusters in the generalized linear model after adjustment for age and sex. Highest levels of Hcy and CRP were present in Cluster A (severe subtype in motor, NMS and cognitive domains), while lowest levels were shown in Cluster C (mild subtype and young age of onset). The differences of Hcy and CRP levels among three cluster were remained significant after adjustment for multiple comparisons. Mean levels of Hcy among three clusters were: 19.4 ± 4.2, 18.4 ± 5.7, 15.6 ± 5.6, *p* = 0.001, *q* = 0.005; while the mean levels of CRP were: 2.5 ± 5.0, 1.5 ± 2.4, 0.9 ± 2.1, *p* = 0.000, *q* < 0.0001 (Table 5). The comparison of Hcy and CRP levels among three clusters remained significant after adjustment for age of diagnosis, sex and significant comorbidities including hypertension, hyperlipidemia, lipid medication and hypertension medication. Please refer to Supplementary Table 2.

**Table 5:** Comparison of blood biomarkers among the three PD clusters.

Blood biochemical markers	Cluster A	Cluster B	Cluster C	*p* value*	*q* value**
	severe cluster	intermediate	mild cluster		
	*n* = 43	*n* = 98	*n* = 65		
Hcy (u/molL)	19.4 ± 4.2	18.4 ± 5.7	15.6 ± 5.6	0.001	0.005
CRP (mg/L)	2.5 ± 5.0	1.5 ± 2.4	0.9 ± 2.1	0.000	<0.0001
Vit D3(ng/mL)	22.8 ± 7.5	23.2 ± 7.3	21.7 ± 6.7	0.522	0.522
UA(mg/dL)	5.3 ± 1.2	5.0 ± 1.4	4.8 ± 1.5	0.200	0.286
TC(mg/dL)	176.0 ± 37.8	195.1 ± 36.2	189.4 ± 31.5	0.045	0.113
TG(mg/dL)	103.5 ± 29.6	110.5 ± 53.6	93.3 ± 34.1	0.039	0.100
HDL(mg/dL)	56.9 ± 13.6	61.3 ± 15.5	61.4 ± 15.8	0.254	0.300
LDL(mg/dL)	98.6 ± 30.8	111.9 ± 31.7	109.4 ± 26.3	0.189	0.286
APOA1(g/L)	1.3 ± 0.2	1.3 ± 0.2	1.3 ± 0.2	0.270	0.300
APOB(g/L)	0.8 ± 0.2	0.9 ± 0.2	0.8 ± 0.2	0.141	0.282

Mean ± standard deviation

*Generalized linear model was applied to compare the biomarkers against different clusters and adjusted for age of diagnosis, sex.

**False discovery rate (FDR) method was performed and *q* values were calculated to control for multiple testing and the threshold of *q* values was set as 0.1.

Hcy: Homocysteine, *CRP* C-reactive protein, *Vit D3* Vitamin D3, *UA* Uric acid; *TC* Cholesterol; *TG* Triglyceride; *HDL-C* high-density lipoprotein cholesterol, *Apo A1* apolipoprotein A1, *LDL-C* low-density lipoprotein cholesterol, *Apo B* apolipoprotein B.

## Discussion

In this study, 206 early PD patients who were recruited within 1 year from diagnosis were assigned to three clusters by an unbiased data-driven hierarchical cluster analysis: cluster A (severe subtype in motor, NMS and cognitive domains), cluster B (intermediate subtype with cognitive impairment and mild NMS) and cluster C (mild subtype and young age of onset). Despite similar disease durations, the three clusters presented with substantially different clinical features and blood biomarker (genetic markers and biochemical markers) profiles. The significantly different allele frequencies in two SNPs (*Park16 rs6679073* A allele and *SV2C rs246814* T allele), suggest that these may be important genetic biomarkers for PD subtypes. We also found Hcy and CRP to be promising biomarkers to identify the severe PD subtype. These findings contribute to our understanding of PD heterogeneity, especially among Asian PD.

Various PD subtypes have been identified through cluster analysis in previous studies. The diffuse malignant cluster previously reported by Fereshtehnejad in two different studies^4,18^ is most akin to cluster A in our study. Cluster A was severe in all disease domains including motor, NMS and cognition. The underlying mechanism of this severe cluster most likely lies in simultaneous involvement of dopaminergic and non-dopaminergic pathways at an early disease stage^18^.

Previous studies reported that the most critical determinants of PD subtype were UPDRS, cognitive status, RBD, and orthostatic hypotension^4,18^. In our study, cluster A was best defined by MDS-UPDRS part I, II, III scores, cognitive impairment and significant BP drop, suggesting that the most critical drivers for PD subtype in our cohort are consistent with previous reports. However, RBD was not found to be an effective clinical determinant for PD subtyping in our cohort. Our study had a low RBD detection rate, which is likely attributable to the use of RBD1Q to detect RBD rather than use of gold standard overnight polysomnography assessment. In addition, the MCI percentage in our cohort was higher than that in PPMI cohort reported by Weintraub^19^. Older age of diagnosis, lower education year and different ethnic population in our cohort may contribute to the difference. The identification of the severe cluster and its critical clinical drivers would enable clinicians to identify PD patients with a more severe subtype at an early disease stage.

Besides the severe cluster, there were two comparatively more benign PD clusters in our cohort. Cluster C was characterised by young onset with generally better performance in all disease domains. This finding is consistent with previous studies^5,20^ that have identified a mild PD subtype with young onset.

Another comparatively benign PD cluster in our cohort was cluster B, comprising 47.6% of the PD patients, with the key features of cognitive impairment and mild NMS. Cluster B is a unique subtype in our cohort with the cognitive performance and NMS scores found to be in opposing directions from each other. The mechanism of cognitive impairment in PD is not fully understood. Acetylcholine neurotransmitter dysfunction is one of the possible pathways^21^. Muller et al reported that cognitive impairment alone in PD patients was related to isolated cortical cholinergic deficits, while a combination of cognitive decline, falls and RBD correlated with thalamic and cortical cholinergic deficiency^22^. The features of cluster B in our cohort suggest that the underlying affected brain areas of cognitive impairment in PD patients might be heterogenous.

When characterizing genetic markers in the three PD subtypes, we found that they had significantly different allele frequencies in two SNPs even though the composite genetic score was not significantly different among three clusters. The mild cluster had significantly higher frequencies of the Park16 rs6679073 A allele and SV2C rs246814 T allele, indicating that these two SNPs may have potential neuroprotective effects in our Asian cohort.

*Park16* SNPs has been consistently reported to play a protective role of PD development in different populations^23,24^. However, there is little information about the clinical characteristics of *Park16* carriers. Our study found that the number of *Park16* carriers was highest in the mild cluster (*Park16* rs6679073 A allele frequency in mild cluster was similar with the healthy control group, results not shown), indicating that *Park16* carriers were likely to have mild symptoms, which supports the SNP’s neuroprotective effects in PD. The underlying mechanism is not entirely clear. One possible explanation may lie in the interaction between *Park16* and *LRRK2*^25^. MacLeod et al. reported that deficiency of the *Park16* locus gene *RAB7L1(*RAB29*)* resulted in neurodegeneration in *LRRK2* mutant neurons while overexpression of RAB7L1 restored the function of neurons with *LRRK2* mutation in an animal model^25^. Future clinical studies are needed to further elucidate the interaction between *Park16 and LRRK2*.

Recently, Foo et al.^9^ reported that synaptic vesicle glycoprotein 2C (SV2C) was a novel gene having robust association with PD development in various populations. It was also reported that SV2C was a functional PD candidate gene and an important mediator of dopamine homeostasis. Genetic deletion of SV2C caused a reduction of dopamine release, resulting in a decrease in motor activity^26^. Our findings corroborate the possible neuroprotective effects of SV2C, as the severe cluster had the lowest percentage of SV2C, while the mild cluster had the highest number of the SV2C carriers.

Our characterization of blood biochemical markers and PD clusters found Hcy to be a promising biomarker for the severe PD subtype after adjusting for confounders and multiple comparisons. Previous evidence have found elevated blood Hcy levels to be associated with cognitive impairment in PD patients^9,27^. However, to our best knowledge, Hcy has not been previously reported to be associated with PD severity. The robust relationship between elevated Hcy levels and severe PD subtype may open new strategies for PD treatment. Since the accelerated rate of brain atrophy in the elderly with MCI have been found to be slowed by treatment with homocysteine-lowering B vitamins^28^, it is worth investigating whether PD severity can be ameliorated by adding vitamin supplementation to lower the Hcy levels. In addition to Hcy, we found CRP to be another reliable biomarker for the severe PD subtype, a finding that is in agreeable with a previous report^8^. These findings lend evidence for the existence of an heightened inflammatory state in severe PD subtypes.

Previous studies have reported that lipids have a neuroprotective effect on PD development. However, it is still controversial if lipid markers are associated with specific PD subtypes. We found that clusters with more than 60% MCI incidence (both clusters A and B) had significantly higher TG level, consistent with our previous finding that higher TG levels were related to cognitive impairment^29^. Our results also showed that the severe cluster (cluster A) had lowest TC levels. However, these associations were not significant after adjusting for multiple comparisons. Lawton et al recently reported that the severe motor disease phenotype, poor psychological well-being, and poor sleep subtype was associated with reduced Apo A1 levels^8^. Our study, however, failed to reproduce this association. We were also unable to find out any significant correlation between our PD subtypes and Vitamin D or UA levels.

Our PD clusters were generated from an Asian cohort with all PD patients recruited within 1 year from diagnosis, which ensures that the cluster features were not driven by different disease durations and stages. In addition, we performed cluster analysis by including genetic status, that enabled us to investigate PD heterogeneity at the genetic level. Our study also tries to assess the association between a broad list of blood biomarkers (genetic markers and serum biochemical markers) and PD clusters, which provides comprehensive biological characterization for the newly generated clusters. However, some limitations of the study should be noted. The current study was cross-sectional and blood biomarkers were not monitored overtime. Longitudinal follow-up of these PD subtypes to monitor their biomarkers and disease progression will be needed. Furthermore, this was a single cohort study with limited sample size that requires further validation in other populations.

In summary, we introduced three subtypes of early PD patients in a multi-centre Asian cohort: ‘severe’, ‘intermediate’ and ‘mild young-onset’ subtypes. The severe subtype was associated with significantly lower frequency of Park16 and SV2C alleles; and had significantly higher levels of serum Hcy and CRP. Park16, SV2C, Hcy and CRP may be useful biomarkers to stratify PD patients into disease subtypes. Our findings also shed light on the possible underlying mechanisms that account for PD heterogeneity. This will improve the stratification of PD patients into disease subtypes that will enable more targeted personalised treatment strategies. Further validation of the genetic and biochemical differences between subtypes in larger cohorts and evaluation of their impact on PD progression is warranted.

## Methods

### Participants and enrolment

#### Study population

A total of 206 idiopathic early PD patients defined by National Institute of Neurological Disorders and Stroke (NINDS) diagnostic criteria have been recruited from Early Parkinson’s disease Longitudinal Singapore (PALS) cohort based on the inclusion and exclusion criteria of PALS study protocol^30^. PALS is an ongoing prospective cohort study undertaken to investigate the disease course of early PD patients who were recruited within 1 year of diagnosis.

#### Enrolment

Our study was conducted at two movement disorder outpatient clinics(Singapore General Hospital and Tan Tock Seng Hospital) in Singapore. Our study has been approved by SingHealth Centralized Institutional Review Board (CIRB) with Ref 2019/2433 and written informed consent was provided by all participants.

### Data collection

Comprehensive clinical features (motor, NMS and cognitive domains) and blood biomarkers were collected and used in the study. All clinical assessments were performed while patients were on their PD medications.

#### Clinical assessments

##### Motor manifestations

Movement Disorder Society-Unified Parkinson’s Disease Rating Scale (MDS-UPDRS) Part II score(Motor Aspects of Experiences of Daily Living), part III motor score, tremor score, Postural Instability and Gait Disorder (PIGD) score^31,32^ were used to assess motor performance. The calculation of tremor score was based on MDS‐UPDRS items: 2.10, 3.15a, 3.15b, 3.16a, 3.16b, 3.17a, 3.17b, 3.17c, 3.17d, 3.17e, and 3.18, while the mean of MDS‐UPDRS items 2.12, 2.13, 3.10, 3.11, and 3.12 was PIGD score^32^.

##### NMS

MDS-UPDRS Part I score (Non-Motor Aspects of Experiences of Daily Living) and Non-motor symptom scale (NMSS) total score were used to assess NMS burden; NMSS^33^ consists of 30 items which are grouped into nine domains (cardiovascular domain, sleep/fatigue, mood/apathy, perceptual problems/hallucinations, attention/memory, gastrointestinal, urinary, sexual function and miscellaneous). Olfaction impairment and autonomic failures are included in the miscellaneous domain. Rapid Eye Movement (REM) Sleep Behavior Disorder (RBD), Daytime sleepiness and sleep quality were evaluated by the RBD Single-Question Screen (RBD1Q)^34^ and Epworth sleepiness scale (ESS) respectively. Patient depression and anxiety were assessed by Hospital Anxiety Depression Scale (HADS).

##### Cognitive impairment

Montreal Cognitive Assessment (MoCA) was performed to monitor the overall cognitive change. Comprehensive neuropsychological tests were performed and 5 cognitive domain scores (executive, visuospatial, memory, attention and working memory, language) were calculated by using the average of the standardized score of two neuropsychological tests from the same domain. Specifically, the following cognitive tests were administered to evaluate the cognitive status of the 5 domains: (1) Executive: Frontal Assessment Battery (FAB) total score and Fruit Fluency; (2) Visuospatial: Repeatable Battery for the Assessment of Neuropsychological Status (RBANS) judgment of Line Orientation and Rey-Osterrieth Complex Figure Test (ROCF) copy total score; (3) Memory: Alzheimer Disease Assessment Scale (ADAS)-cog delayed recall score and ROCF delayed recall total score; (4) Attention and working memory: Digit Span Backward and Symbol Span total score; (5) Language: Boston Naming Test (BNT) total score and Wechsler Adult Intelligence Scale | Fourth Edition (WAIS-IV)-Similarities. Mild Cognitive Impairment (MCI) diagnosis was based on International Parkinson and Movement Disorder Society (MDS) level II criteria^35^, in which cognitive impairment should be present in at least two neuropsychological tests with 1.5 standard deviations (SDs) worse than norms as cut offs, either within a single cognitive domain or across different cognitive domains.

##### Others

Blood pressure was measured both in the supine position and after 3 min of standing. Orthostatic drop in Systolic Blood Pressure(SBP) greater than 10 mmHg was considered significant BP drop and viewed as an objective measure of autonomic dysfunction^4^. We also collected demographic data including sex, age of diagnosis, ethnicity.

#### Blood biomarkers assessments

We genotyped variants of SNCA, LRRK2, Park16, ITPKB, SV2C using Illumina Infinium Global Screening Array − 24 v2.0. The PRS was defined as the sum of the number of risk alleles per individual weighted by their effect size estimate corresponding to the logarithm of the odds ratio^36^. In the current study we calculated PRS by comprising 5 Asian GWAS SNPs (*SNCA, LRRK2, Park16, ITPKB, SV2C*) with the highest effect size and p level less than the genome wide significant association level (5*10^−8^) from the latest Asian GWAS meta-analysis^37^ to provide quantitative data of genetic burden individually. The SNPs data being used for PRS calculation can be found in supplementary data.

We tested 10 commercially available blood biomarkers. They are homocysteine (Hcy), C-reactive protein (CRP), vitamin D, uric acid(UA) and lipid markers including Triglyceride (TG), total cholesterol (TC), high-density lipoprotein cholesterol (HDL-C), low-density lipoprotein cholesterol (LDL-C) Apolipoprotein A1 (Apo A1) and Apolipoprotein B (Apo B). Blood biomarkers were measured using overnight fasting venous serum sample and were determined by enzymatic assay in a professional medical laboratory (Quest Laboratories Pte Ltd, Singapore).

### Statistical methods

#### Cluster analysis

Cluster analysis was performed in Python Software version 3 (http://www.python.org). Seventeen variables (Age of diagnosis, PRS, Number of patients having significant BP drop, MDS-UPDRS Part II score, MDS-UPDRS Part III score, tremor score, PIGD score, MDS-UPDRS Part I score, ESS Total Score, HADS Anxiety Total score, HADS Depression Total score, RBD1Q, Memory score, Visuospatial score, Attention score, Language score, Executive score) were selected by expert opinion and contemporary evidence^18^. All variable measurements were standardized by using the Z-scores for the cluster analysis. Agglomerative hierarchical clustering with Euclidean distance calculation was applied. We selected the three-cluster solution due to more balanced data distribution and better clinical interpretation. Missing value pattern was identified as missing by random. Hence, single imputation approach was used to impute 92 (2.6%) missing values in the baseline variables.

#### Post-hoc comparisons of clinical characteristics and allelic distributions of related genes

Post-hoc comparisons were performed in Stata software (Stata/SE 16.1 Stata Corp. 2019. Stata Statistical Software: Release 16. College Station, TX: Stata Corp LLC) and SAS OnDemand for Academics (SAS Institute Inc. 2014. SAS® OnDemand for Academics: User’s Guide. Cary, NC: SAS Institute Inc.). Continuous variables were summarized using mean with standard deviation (SD) or median with first and third quartile. Categorical variables were summarized by frequencies and percentages. Demographics, clinical characteristics not included in cluster analysis and allelic distributions of related PD genes were compared among clusters. Fisher’s exact test or Pearson Chi square test (where appropriate) was carried out to compare the categorical variables among different clusters; while one-way ANOVA or Kruskal-Wallis tests (depends whether normality assumption was tenable) was performed to compare continuous variables among different clusters.

#### Blood biochemical markers comparisons among clusters

Blood biochemical markers comparisons were performed in SAS OnDemand for Academics (SAS Institute Inc. 2014. SAS® OnDemand for Academics: User’s Guide. Cary, NC: SAS Institute Inc.). All blood biochemical markers except CRP were log-transformed to reduce the right-skewness. Generalized linear model was performed to compare the biomarkers among different clusters and adjusted for age of diagnosis, sex, using normal distribution assumption for the outcome variable. Gamma distribution was assumed for CRP due to the skewed distribution even after log-transformation. False discovery rate (FDR) method^38^ was performed to control for multiple testing comparison and q value was calculated. We set the threshold of *q* values as 0.1.

## Supplementary information

Supplementary Tables

Supplementary Data

## Data Availability

The data collected during this study are available from the corresponding author upon reasonable request from qualified individuals.

## Code availability

No previously unreported custom computer code or algorithm was used to generate results in this study.
