# Supplementary material for: Biomarker characterization of clinical subtypes of Parkinson Disease
Source: NPJ Parkinsons Dis. 2022 Aug 29;8:109. doi: 10.1038/s41531-022-00375-y (PMC9424224; doi:10.1038/s41531-022-00375-y)
Supplement: Supplementary file 1 — Supplementary Tables [file 41531_2022_375_MOESM1_ESM.pdf]

Supplementary Table 1: Comparison of the comorbidities among the three PD clusters

Comorbidities and treatment	cluster A severe cluster N=43	cluster B intermediate N=98	cluster C mild cluster N=65	p-value*
BMI (kg/m^2)	23.83±3.37	23.36± 3.26	22.36± 3.10	0.440
DM (%)	12 (28%)	15 (15%)	8 (12%)	0.10
Hypertension (%)	29 (67%)	46 (47%)	20 (31%)	<0.001
Hyperlipidaemia(%)	28 (65%)	47 (48%)	22 (34%)	0.006
Gout (%)	2 (5%)	3 (3%)	3 (5%)	0.72
DM medication (%)	11 (26%)	15 (15%)	8 (12%)	0.18
Lipid medication (%)	28 (65%)	46 (47%)	19 (29%)	0.001
Hypertension medication (%)	29 (67%)	47 (48%)	18 (28%)	<0.001

Abbreviations: BMI: Body Mass Index; DM: Diabetes Mellitus
Categorical variables reported as frequency (%)
* *Chi-square or Fisher exact test (where appropriate) for categorical variables*

Supplementary Table 2: Comparison of key blood biomarkers among the three PD clusters

Blood biochemical markers	cluster A severe cluster n=43	cluster B intermediate n=98	cluster C mild cluster n=65	p value*
Hcy (u/molL)	19.4 ± 4.2	18.4 ± 5.7	15.6 ± 5.6	0.01
CRP (mg/L)	2.5 ± 5.0	1.5 ± 2.4	0.9 ± 2.1	0.0004

** Generalized linear model was applied to compare the biomarkers against different clusters and adjusted for age of diagnosis, sex and significant comorbidities including hypertension, hyperlipidaemia, lipid medication and hypertension medication.*
